# Growth and Airborne Transmission of Cell-Sorted Life Cycle Stages of *Pneumocystis carinii*

**DOI:** 10.1371/journal.pone.0079958

**Published:** 2013-11-06

**Authors:** Anna Martinez, Marie C. M. Halliez, El Moukhtar Aliouat, Magali Chabé, Annie Standaert-Vitse, Emilie Fréalle, Nausicaa Gantois, Muriel Pottier, Anthony Pinon, Eduardo Dei-Cas, Cécile-Marie Aliouat-Denis

**Affiliations:** 1 Biology and Diversity of Emerging Eukaryotic Pathogens (BDEEP), Center for Infection and Immunity of Lille (CIIL), INSERM U1019, CNRS UMR 8204, EA-4547, Univ Lille Nord de France, Institut Pasteur de Lille, Lille, France; 2 Japan Collection of Microorganisms, RIKEN BioResource Center, Koyadai, Tsukuba, Ibaraki, Japan; 3 Biological sciences department, Inflammation research network, University of Calgary, Calgary, Alberta, Canada; 4 Unité de Sécurité Microbiologique, Institut Pasteur de Lille, Lille, France; 5 Centre Régional Hospitalier Universitaire, Biology and Pathology Center, Parasitology-Mycology, Lille, France; University of Heidelberg Medical School, Germany

## Abstract

*Pneumocystis* organisms are airborne opportunistic pathogens that cannot be continuously grown in culture. Consequently, the follow-up of *Pneumocystis* stage-to-stage differentiation, the sequence of their multiplication processes as well as formal identification of the transmitted form have remained elusive. The successful high-speed cell sorting of trophic and cystic forms is paving the way for the elucidation of the complex *Pneumocystis* life cycle. The growth of each sorted *Pneumocystis* stage population was followed up independently both in nude rats and *in vitro*. In addition, by setting up a novel nude rat model, we attempted to delineate which cystic and/or trophic forms can be naturally aerially transmitted from host to host. The results showed that in axenic culture, cystic forms can differentiate into trophic forms, whereas trophic forms are unable to evolve into cystic forms. In contrast, nude rats inoculated with pure trophic forms are able to produce cystic forms and *vice versa*. Transmission experiments indicated that 12 h of contact between seeder and recipient nude rats was sufficient for cystic forms to be aerially transmitted. In conclusion, trophic- to cystic-form transition is a key step in the proliferation of *Pneumocystis* microfungi because the cystic forms (but not the trophic forms) can be transmitted by aerial route from host to host.

## Introduction

Opportunistic fungal organisms, belonging to the *Pneumocystis jirovecii* species, are responsible for a severe interstitial lung disease, called *Pneumocystis pneumonia* (PcP), that can occur in immunocompromised patients and is fatal without effective treatment [1,2]. In addition to *P. jirovecii*, the fungal genus *Pneumocystis* encompasses a number of host-specific species that infect a wide range of mammals [3]. Studies of PcP in murine models have led to a better understanding of the pathogenesis and transmission of *Pneumocystis* organisms [4-7]. During PcP, *Pneumocystis* organisms filling the alveolar space mostly consist of trophic forms, whereas sporocytes and mature cysts are a minority [8]. Although the main transmission route has been clearly established as being aerial [9,10],, the life cycle stage responsible for disease transmission has not been formerly proven. *Pneumocystis*-infected mice treated with anidulafungin, an echinocandin drug inhibiting the formation of the cyst cell wall, were previously shown to be unable to transmit the infection to susceptible mice [11], thus suggesting a major role of the cysts in *Pneumocystis* transmission. In the present study, the life cycle of *P. carinii* is further dissected by independently following the growth kinetics of trophic and cystic forms both in axenic culture and *in vivo* after endotracheal infection of nude rats. To further determine the transmission form of PcP, a novel nude rat model of natural infection was specifically established.

## Results

### Growth of sorted *P. carinii* trophic and cystic populations in axenic culture

**Figure 1:**
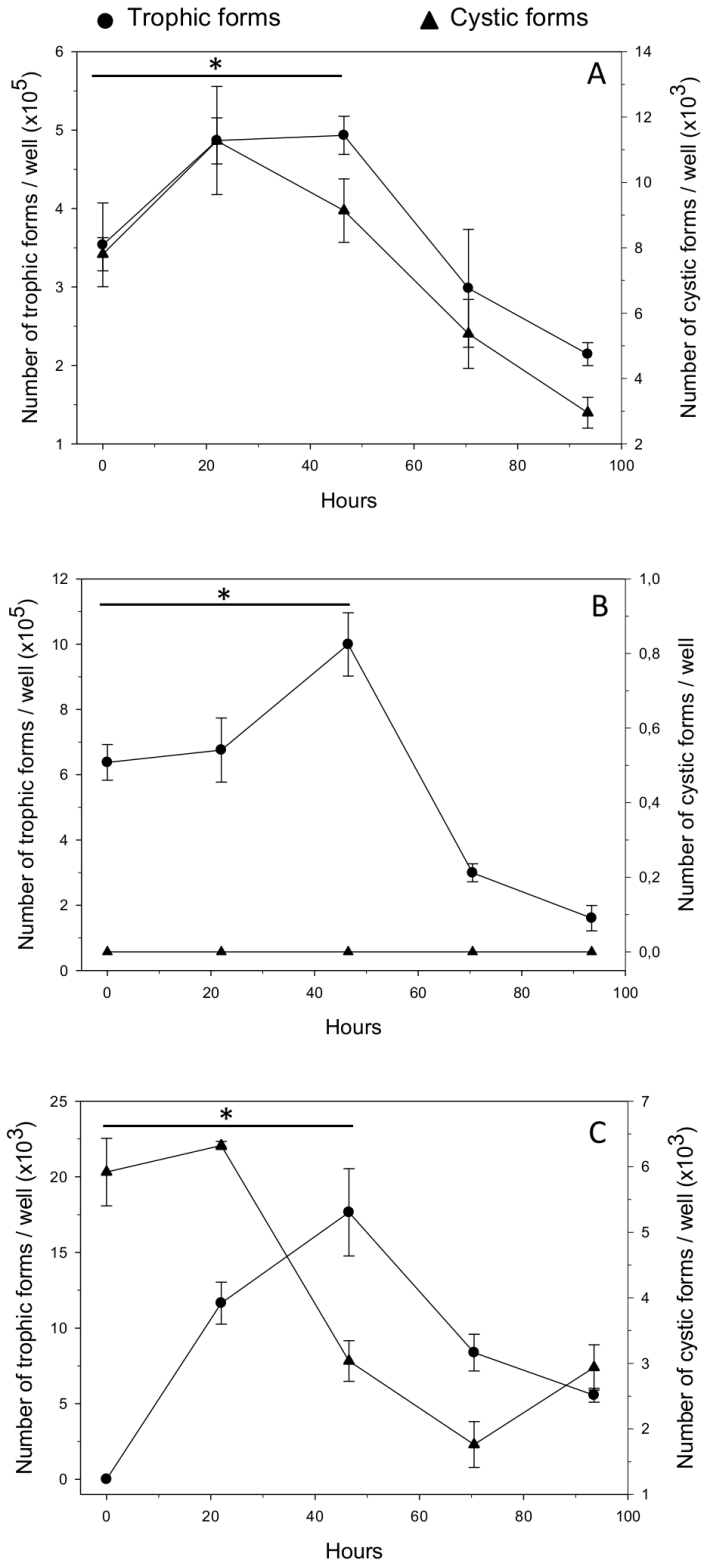
Growth of sorted *P. carinii* organisms in axenic culture. Several *P. carinii* populations were cultured in DMEM with 10% FBS, at 37°C, in an atmosphere containing 5% CO_2_. Growth of (A) sorted *P. carinii* total population devoid of host cell debris, (B) pure *P. carinii* trophic forms, and (C) pure *P. carinii* cystic forms is followed during 4 days. Trophic forms (circles, left Y-axis) or cystic forms (triangles, right Y-axis) were microscopically quantified after RAL-555 panoptic staining [26-28]. For each population of *P. carinii* organisms studied, means of three replicates are represented per time point. Error bars represent standard deviations. The star (*) means *P*-value ≤ 0.05. The detection limit is 10 *P. carinii* organisms per culture well.

Growth of either trophic or cystic forms were followed *in vitro* for (i) a sorted *P. carinii* total population devoid of host cell debris, (ii) pure *P. carinii* trophic forms, or (iii) pure *P. carinii* cystic forms. Fungal cultures have been followed for 4 days but growth stopped after 2 days and parasite numbers decreased (Figure 1). When total sorted *P. carinii* organisms were cultured for 2 days, the number of trophic forms increased (*P* = 0.05, Figure 1A), while the increase in cystic forms was less pronounced. When cultured alone (Figure 1B), trophic forms grew significantly until day 2 (*P* = 0.05). Strikingly, no detectable *de novo* production of cystic forms occurred when the culture was set up with pure trophic forms (Figure 1B). No cysts were detected even after 4 days of culture. Conversely, pure cystic forms were able to release trophic forms during the 2 days of culture (Figure 1C). Indeed, the number of cystic forms abruptly decreased by day 2 (*P* = 0.05), while the number of trophic forms, starting from below the detection limit (10 *P. carinii* organisms per culture well), grew quickly (*P* = 0.037).

### Differentiation of sorted *P. carinii* organisms in nude rats

**Figure 2:**
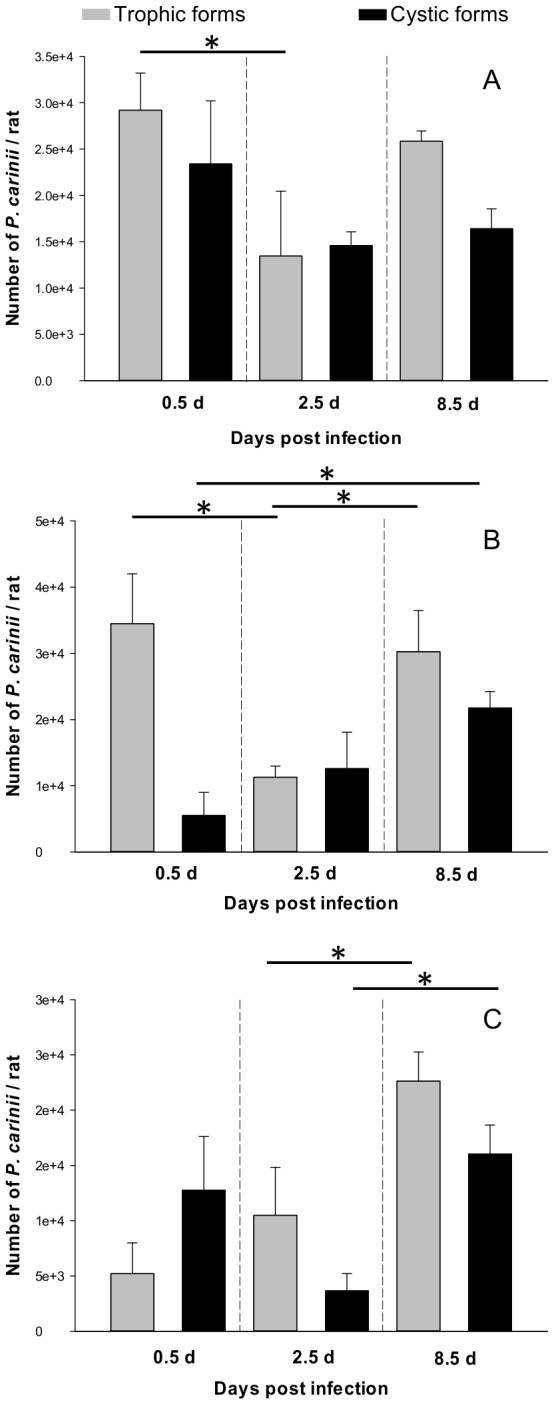
Infection of nude rats with sorted *P. carinii* organisms. Nude rats were endotracheally infected with three *P. carinii* sorted populations: (A) sorted *P. carinii* total population devoid of host cell debris, (B) pure *P. carinii* trophic forms, and (C) pure *P. carinii* cystic forms. Rats were euthanized at 0.5 (12 h), 2.5 days, and 8.5 days postinoculation and then parasites were extracted from lungs [29]. After staining with RAL-555, trophic forms (in grey) or cystic forms (in black) were microscopically quantified [26-28]. At each time point, means of either trophic- or cystic-form burdens developing in the lungs of three animals are plotted on the same Y-axis. Error bars represent standard deviations. The star (*) means *P*-value ≤ 0.05.

When nude rats were infected with total sorted *P. carinii* organisms, a significant (*P* = 0.05) decrease in the number of the trophic forms was obvious over the first 2.5 days following endotracheal infection (Figure 2A). At 8.5 days postinfection, this number tended to increase. Conversely, the cystic population remained stable during the course of the experiment (Figure 2A). Growth kinetics of trophic forms were similar in nude rats infected with pure trophic forms (Figure 2B). Indeed, following a decrease in the number of trophic forms (*P* = 0.05, Figure 2B), their development significantly resumed on day 8.5 (*P* = 0.034). Interestingly, cystic forms were produced as early as 12 h postinfection and their number increased significantly until the end of the experiment (*P* = 0.034, Figure 2B). Finally, when nude rats were infected with pure cystic forms, trophic forms were detected as early as 12 h after infection (Figure 2C). These forms actively and steadily increased thereafter (*P* = 0.05). The cystic-form burden significantly increased from day 2.5 until the end of the experiment (*P* = 0.05, Figure 2C).

### Airborne transmission of *P. carinii*-stage populations to nude rats

The aim of this experiment was to decipher which of the trophic and/or cystic forms are important for *P. carinii* aerial transmission. The choice of a short contact time (12 h) between seeder and receiver animals was dictated by the finding that trophic and cystic forms differentiate quickly once in the rat lungs (Figures 2B and C). 

At the end of the contact period, all seeder rats were found to harbor a substantial burden of *P. carinii* organisms (Table 1). Interestingly, when molecular detection by nested-PCR (nPCR) was performed, only receiver rats that were placed in contact with seeder rats infected with pure *P. carinii* trophic forms were negative for the presence of *P. carinii* organisms (Table 1). Seeder rats infected with either sorted *P. carinii* total population or pure cystic forms were able to transmit *P. carinii* organisms to three out of four receiver rats within a contact period as short as 12 h. Nevertheless, once immunosuppressed for 7 weeks, none of these receiver animals were prone to PcP development (Table 1). Whole lung tissue extracts of sentinel noninfected nude rats were all negative for the presence of *P. carinii* gDNA.

**Table 1:** Airborne transmission of *P. carinii* stage populations to nude rats.

	**Detection of *P. carinii* organisms** (at the end of contact)			**PcP development** (7 weeks postcontact)
	**Seeder rats**		**Receiver rats**	**Receiver rats**
***P. carinii*-stage population inoculated to seeder rats**	**Mean *P. carinii* burden** ^(1)^	**Molecular detection** ^(2)^	**Molecular detection** ^(3)^	**Microscopic and molecular detection** ^(4)^
**Pure *P. carinii* total population**	6.2 × 10^3^ ± 2.48 × 10^3^ (*n* = 19)	All positive (*n* = 19)	3 positive (*n* = 4)	All negative (*n* = 4)
**Pure *P. carinii* trophic forms**	8.9 × 10^3^ ± 4.9 × 10^3^ (*n* = 19)	All positive (*n* = 19)	All negative (*n* = 4)	All negative (*n* = 4)
**Pure *P. carinii* cystic forms**	3.9 × 10^3^ ± 2.0 × 10^3^ (*n* = 16)	All positive (*n* = 16)	3 positive (*n* = 4)	All negative (*n* = 4)

(1) *P. carinii* burden is microscopically quantified as the number of trophic forms and/or cystic forms on calibrated RAL-555 stained-smears (2). A single-round touchdown PCR (TD-PCR) targeting the multicopy gene coding for the ribosomal RNA of the large mitochondrial subunit (mtLSUrRNA) was used to detect *P. carinii* gDNA in lung tissue extracts of seeder rats [32]. (3) Since TD-PCR at the mtLSUrRNA locus revealed to be negative for receiver rats, nested-PCR (nPCR) at the same locus was subsequently performed on whole lung tissue extract to increase sensitivity [5]. (4) Following 7 weeks of immunosuppression, PcP development was microscopically monitored on smears of lung tissue extracts stained with RAL-555 [26-29]; as careful microscopical screening revealed to be negative, nPCR at the mtLSUrRNA was performed on all lung tissue extracts. Whole lung tissue extracts of all sentinel rats (*n* = 6) were also screened for the presence of *P. carinii* gDNA by nPCR at the mtLSUrRNA locus and all samples were negative. The number (n) of individual rats analyzed is indicated for each animal group.

## Discussion

**Figure 3:**
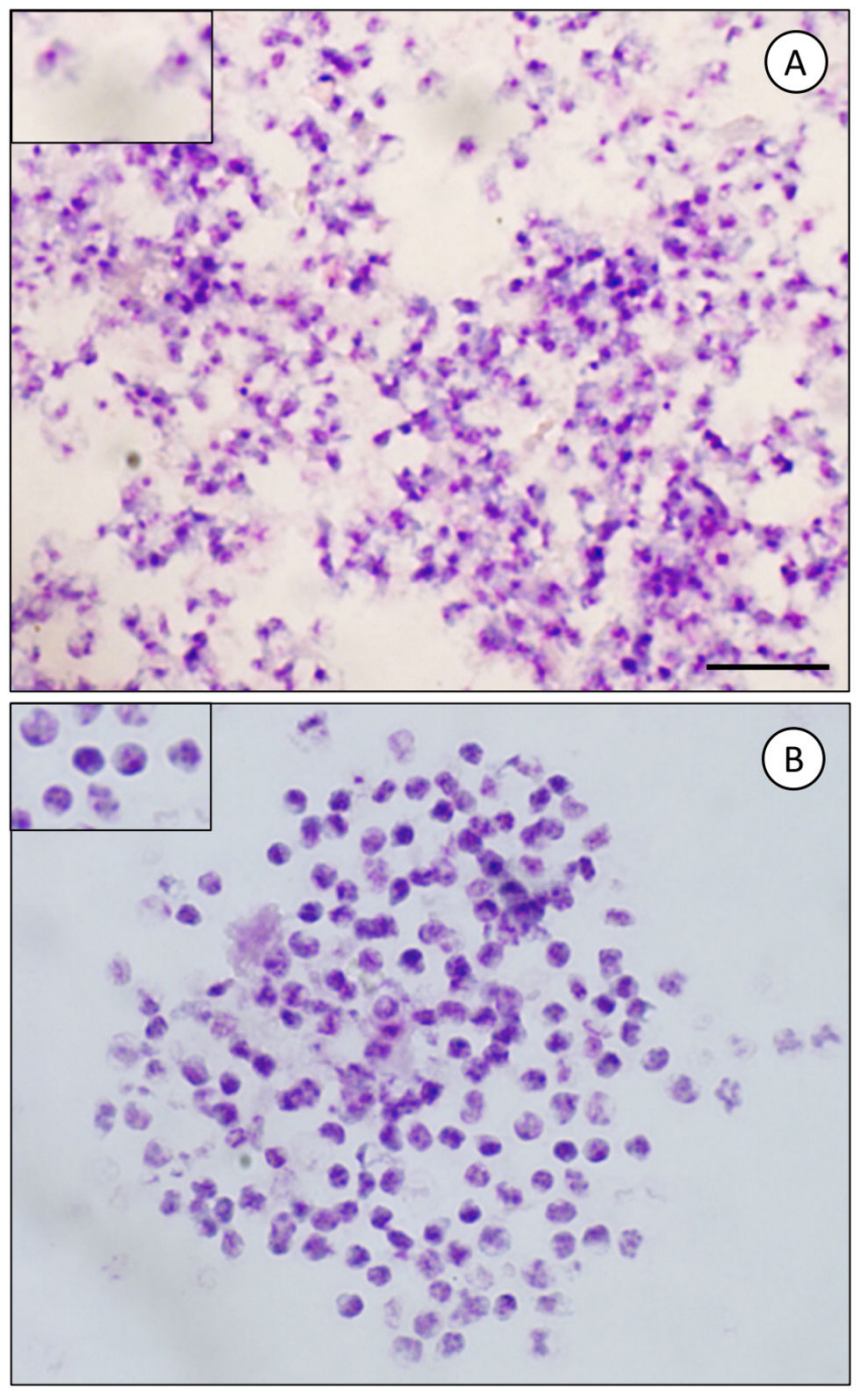
Purity of sorted *P. carinii* cystic and trophic forms. *P. carinii* cyst forms were physically separated from trophic forms by cell sorting using high-speed flow cytometry [12]. To check for purity, smears of sorted *P. carinii* organisms were stained with RAL-555, a panoptic Giemsa-like stain. No cystic forms were noted in the sorted trophic-form fraction (A). No trophic forms were visible within the sorted cystic-form fraction (B). *P. carinii* cell sorting was reproducible, reaching 99.6% purity [12]. Insets represent a higher magnification. Bars = 10 μm.

To date, no continuous culture model allows the maintenance of *P. carinii* organisms, thus impairing the follow-up of stage-to-stage differentiation of this still enigmatic fungus. An attempt to further elucidate its complex life cycle was to physically separate *P. carinii* life cycle stages with a thin cell wall (herein called trophic forms) from those with a thick cell wall (called cystic forms). High-speed cell sorting has allowed the purification of infectious *P. carinii* life cycle stages with high reproducibility and purity (Figure 3) [12]. In the present study, we first aimed at elucidating how trophic and cystic forms contribute to the overall *P. carinii* growth both *in vitro* and *in vivo*. The second aim was to evaluate whether trophic, cystic, or both forms could be aerially transmitted from seeder to receiver hosts and whether these forms could elicit PcP.

First, cultivation of pure trophic forms did not lead to a detectable *de novo* production of cystic forms, while a significant increase in the number of trophic forms occurring at day 2 attested to their dividing capacity (Figure 1B). Conversely, when only cysts had been cultured, trophic forms were detected as early as day 2 while the number of cysts decreased (Figure 1C). The rapid occurrence of trophic forms in our model can be explained by the presence, in the starting inoculum, of mature cysts, ready to rupture and release spores that evolve toward young trophic forms. The production of trophic forms from cystic forms has already been postulated before on the basis of growth kinetics [13] or ultrastructural observations [14]. Trophic forms could subsequently initiate mitotic divisions, a quick way to boost growth, as hypothesized before [15]. Within 48 h, a decrease in the number of cystic forms concomitant to an increase in the number of trophic forms support this hypothesis (Figure 1C). Overall, these results suggest that, once cysts are formed, maturation and/or excystation can rapidly proceed *in vitro* and lead to the *de novo* production of trophic forms that are able to mitotically divide. In contrast, a blocking step seems to prevent *de novo* production of cystic forms *in vitro* when only trophic forms are incubated. This phenomenon could be related to the lack of extrinsic mating inducer signals *in vitro*. It also suggests that trophic-to-cystic form differentiation is an essential step in the turnover of the *P. carinii* life cycle, which may be one of the reasons why the *P. carinii* organisms do not grow continuously in culture [16,17].

The overall shape of the trophic forms’ growth kinetics in animals infected with either sorted *P. carinii* total population or pure trophic forms were similar (Figures 2A and B). Both populations showed a decrease in number in the first days of growth and a boost in the parasite growth towards the end of the follow-up period. Even if inflammation is reduced in nude rats treated with dexamethasone, the initial decrease could be related to the clearance of *P. carinii* organisms due to phagocytosis by macrophages and neutrophils. This phenomenon has already been described in the Scid mice model [18]. What is most striking, in contrast to *in vitro* studies, is the early detection of cystic forms in animals infected with trophic forms only (Figure 2B). Based on what is known about the *P. carinii* life cycle [17,19-21], two hypotheses could explain this finding: (i) thin-walled early sporocytes may be present in the starting inoculum and may be able to differentiate into thick-walled sporocytes within a short period of time and (ii) the mating between trophic forms and subsequent maturation to early and intermediate sporocytes may occur quite rapidly after inoculation. Although cystic forms appeared early in trophic-form-infected animals, their number increased rather slowly but steadily (Figure 2B). This could be due to (i) low mating frequency between trophic forms and/or (ii) a long period of time necessary for sporocytes to evolve towards mature cysts. Another reason why cystic forms cannot accumulate is related to their rupture once they are fully mature. The subsequent spore release can contribute to the late increase in the number of trophic forms, which probably also augments as a result of the mitotic divisions of these forms. When nude rats are solely infected with *P. carinii* cystic forms, the growth kinetics are drastically different: (i) the presence of trophic forms was rapidly detectable, at 12 h postinfection, and their number constantly increased up to day 8.5 and (ii) the cystic forms multiplied from day 2.5 until the end of the experiment (Figure 2C). In these animals, both cystic and trophic forms reached an approximately comparable organism burden as in animals infected with the total sorted *P. carinii* population or trophic forms only. The smaller cyst inoculum may elicit less clearance by the rat innate immune system and is sufficient to initiate active growth [22]. The rapid initial occurrence of trophic forms was probably consecutive to the spore release from mature cysts, whereas the significant increase observed at the end of the follow-up period could be a consequence of mitotic divisions.

Our novel natural aerial transmission model indicates that animals infected with *P. carinii* trophic forms were unable to transmit the fungal organisms to any of the receiver nude rats after 12 h of co-housing (Table 1). In contrast, animals infected with *P. carinii* total population or cystic forms-only, were able to seed the fungal organisms to co-housed receiver animals. Our data further strengthen the notion that cystic forms are the transmitted stage and agree with [11], who, using echinocandins, indirectly showed that these forms were important in the transmission process. Although *P. carinii* DNA was detected in the lungs of positive recipient nude rats, none of them developed PcP at the end of the immunosuppression period. Several hypotheses may account for this unexpected finding: (i) the contact period may have been too short to allow enough organisms to be seeded, (ii) the infectivity of sorted *P. carinii* is lower than nonsorted organisms, although they were shown to be able to elicit PcP [12], and (iii) the innate immune response occurring in seeder rats may be responsible for a drastic reduction in the number of inoculated organisms, thus diminishing the number of exhaled organisms.

To conclude, the growth of *Pneumocystis* cystic and trophic form populations has been followed up independently, both *in vivo* and *in vitro*, for the first time. The trophic-to-cystic form transition appears as a key step in the proliferation and the life cycle of *Pneumocystis* microfungi. Only the cystic forms can be transmitted by aerial route from host to host [11,the current study]. Thus, producing cystic forms by mating inside its mammalian host appears to be the only way for *Pneumocystis* to perpetuate its life cycle from host to host, as no environmental form has been evidenced so far. This is a unique trait amongst aerially transmitted ascomycetes. 

## Materials and Methods

### Ethics statement

All animal experiments were conducted following the guidelines of the Pasteur Institute of Lille animal study board, which conforms to the Amsterdam Protocol on animal protection and welfare, and Directive 86/609/EEC on the Protection of Animals Used for Experimental and Other Scientific Purposes, updated in the Council of Europe’s Appendix A (http://conventions.coe.int/Treaty/EN/Treaties/PDF/123-Arev.pdf). The animal work also complied with the French law (nu 87-848 dated 19-10-1987) and the European Community’s 1976 Amendment of Cruelty to Animals Act. The animal house (accreditation number: A59107, agreement number: B 59-350009) was placed under the direct control of the director of the Pasteur Institute of Lille, who is the “designated responsible person” under French law. The experiment protocol used in the present study has been approved by the Ethics Committee for Experiments on Animals of the Nord-Pas-de-Calais region (approval number ECEA 022011) and was carried out by qualified personnel.

### Source of *Pneumocystis carinii*

Athymic *Pneumocystis*-free Lou nu/nu rats (Pasteur Institute of Lille, France) were used as the source of *Pneumocystis carinii* organisms for all experiments [12]. Briefly, nude rats were administered dexamethasone (Merck Sharp & Dohme Chibret) starting from 2 weeks prior to nonsurgical endotracheal infection with cryopreserved *P. carinii* organisms [12,23]. All animal groups were housed in independent compartments in HEPA-filtered air isolators (Flufrance, Wissous, France) and were provided with sterile irradiated food (Scientific Animal Food & Engineering (SAFE), Augy, France) and sterile water *ad libitum*. Eight weeks postinfection, all animals harbored a high *P. carinii* burden and were euthanized. Their lungs were dissected to isolate organisms that were cryopreserved while remaining highly infectious [12,24,25].

### Cell sorting of *P. carinii* cystic and trophic forms

To study growth kinetics and transmission of *P. carinii* trophic and cystic forms, high-speed cell sorting of *P. carinii* lifecycle-stage populations was performed [12]. Two parasite-stage fractions can be sorted simultaneously: (1) the trophic-form fraction corresponds to parasite stages bearing a thin cell wall (trophic forms, early sporocytes) and (2) the cystic-form fraction includes parasite stages possessing a thick cell wall (intermediate-to-late sporocytes, and mature cysts). The purity of cystic *versus* trophic forms was checked by microscopy prior to any subsequent experiments and was higher than 99.6% (Figure 3) [12]. Sorted *P. carinii* organisms were also shown to remain infectious for the nude rat [12].

### Growth of sorted *P. carinii* organisms in axenic culture

The growth of (i) sorted *P. carinii* total population devoid of host cell debris, (ii) pure *P. carinii* trophic-form fraction, or (iii) pure *P. carinii* cystic-form fraction were followed *in vitro*. Axenic short-term cultures of *P. carinii* were carried out in 24-well plates (Costar) as described in [26] with modifications as mentioned hereafter. Either 5 × 10^5^ *P. carinii* organisms (total population or trophic-form fraction) or 1.5 × 10^4^ *P. carinii* of the cystic-form fraction were suspended in 2 mL of DMEM supplemented with antibiotics (100 U/mL penicillin; 100 µg/mL streptomycin; Sigma-Aldrich) and 10% heat-inactivated fetal bovine serum (FBS, GIBCO BRL, Life Technologies Inc.). *P. carinii* organisms were incubated for 4 days in 5% CO_2_ at 37°C [26]. Every day, organisms from each well were re-suspended in 50 µL of Dulbecco phosphate buffer solution (DPBS, Sigma-Aldrich). The organisms were quantified by microscopic numeration as previously described [26-28]. All experiments were conducted in triplicate.

### Infection of nude rats with sorted *P. carinii* organisms

In order to monitor *P. carinii* growth *in vivo*, dexamethasone-treated nude rats were endotracheally infected with sorted *P. carinii* organisms [12,23]. Three groups of nine animals were infected with three different *P. carinii* populations as follows: (i) 1.3 × 10^6^ cells per animal of sorted *P. carinii* total population (trophic and cystic forms), devoid of host cell debris, (ii) 1.3 × 10^6^ pure trophic forms per animal, and (iii) 3 × 10^5^ pure cystic forms per animal. Three animals per group were euthanized at 12 h, 2.5 days, and 8.5 days postinfection. In order to accurately evaluate parasite burdens, infected lungs were homogenized using a stomacher (Stomacher 80 Biomaster Lab Blender, Seward) as previously described [29]. The resulting pellets were then suspended in DPBS and all *P. carinii* forms were microscopically quantified as described previously [26-28].

### Nude rat model of airborne *P. carinii* transmission

**Figure 4:**
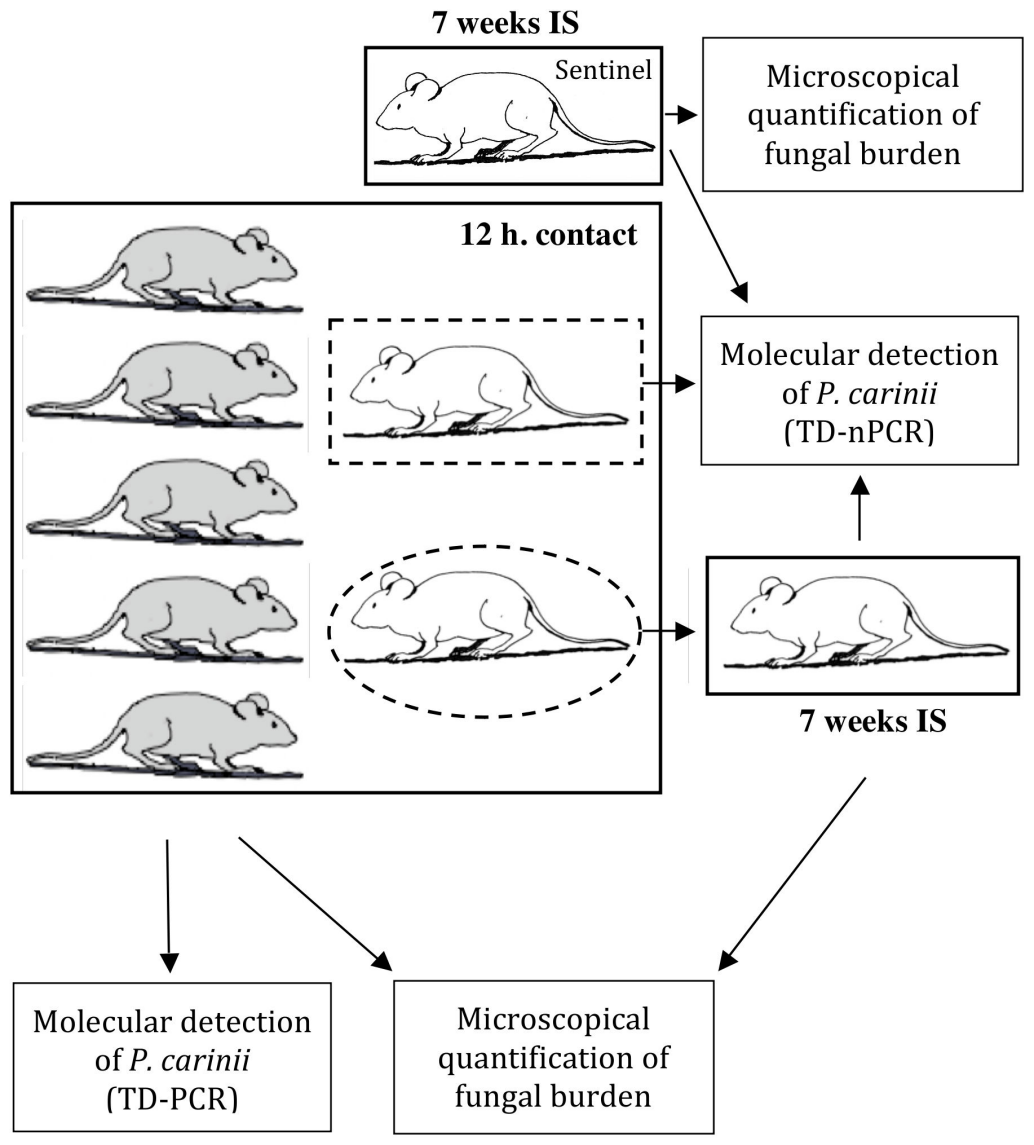
Nude rat model of aerial *P. carinii* transmission. To study the airborne transmission of *P. carinii*, three groups of animals were infected with either *P. carinii* total population or trophic or cystic forms. To ease comprehension, only a portion (1/4) of the animals belonging to a single group is represented on the figure. First, seeder rats (in grey) were endotracheally infected with *P. carinii* organisms (total sorted *P. carinii* population, pure trophic forms, or cystic forms) [23]. Second, after 15 min of recovery, seeder rats were placed in close contact with receiver animals (in white) for 12 h at a mean ratio (seeders to receivers) of 2.4 per cage. These dexamethasone-treated animals were co-housed in capped cages within an individual compartment (thick black square line) of an HEPA-filtered air isolator. Third, all the seeder and half of the receiver rats were euthanized at the end of the contact period. *P. carinii* organisms were extracted from the seeder rat lungs [29]: the fungal burden was microscopically quantified [26-28] and molecular detection of the *P. carinii* mtLSUrRNA gene was performed using a single-round touchdown PCR (TD-PCR, [32]). Whole lung tissue suspensions of half of the receiver rats (dotted square line) were screened by nested-PCR (nPCR) at the same locus to detect *P. carinii* gDNA [5]. Fourth, the other half of the receiver rats (dotted oval line) were kept under immunosuppression (IS) in HEPA-filtered air conditions for 7 weeks to microscopically monitor for eventual PcP development. nPCR was also performed on whole lung tissue suspensions to detect *P. carinii* gDNA. Whole lung tissue suspensions of sentinel rats were also screened by nPCR and by microscopy after 7 weeks of immunosuppression.

By setting up a novel nude rat model of airborne transmission of *P. carinii* organisms (Figure 4), we attempted to approach the natural conditions of transmission in order to identify the *P. carinii* airborne-transmitted form. Three groups of 16–19 immunosuppressed nude rats were endotracheally infected with (i) 6.45 × 10^6^ *P. carinii* per rat from the total sorted population, (ii) 12 × 10^6^ pure trophic forms per rat, or (iii) 9.35 × 10^4^ pure cystic forms per rat [12,23]. These animals, called seeder rats, were allowed to recover from inoculation for 15 min and, then, were housed with eight dexamethasone-treated *P. carinii*-free nude rats, called receiver rats, for 12 h (Figure 4). The mean seeder-to-receiver rat ratio was 2.4 per cage. At the end of contact, all seeder and four receiver rats were euthanized. The four remaining receiver rats were kept separately under dexamethasone treatment for 7 weeks and then euthanized. Two sentinel noninfected nude rats were used as internal negative controls for each of the three groups of animals. After 7 weeks of immunosuppression, they were also euthanized. Each group of animals was kept in a separate compartment of an HEPA-filtered isolator to avoid any risk of contamination. The lungs of all rats were aseptically removed before parasite extraction [29]. Lung tissue suspensions were then screened for the presence of *P. carinii* genomic DNA (gDNA), either by single-round touchdown PCR (TD-PCR) in seeder rats or nested-PCR (nPCR) in receiver and sentinel rats. Lung tissue suspensions from receiver and sentinel rats were also microscopically monitored to detect PcP development 7 weeks postcontact [26-28].

### Molecular detection of *P. carinii* genomic DNA

For seeder rats, a single gDNA extraction was conducted starting from 25 mg of *P. carinii*-infected lung tissue using the tissue protocol of the QIAmp DNA MiniKit (QIAGEN). gDNA was eluted twice in Tris-EDTA (TE) buffer as recommended. For receiver and sentinel rats, since lower quantities of *P. carinii* gDNA were expected, gDNA was extracted from *P. carinii*-infected whole lungs following the tissue protocol of the NucleoBond^®^ AXG 500 kit (Macherey Nagel). Ten gDNA eluates were collected, precipitated, and centrifuged at 20,000 *g*. The pellets were washed with 70% ethanol and then dissolved (55°C for 2 h.) in 500 µL of TE buffer (pH 8.0). Three series were needed to extract gDNA from a whole lung. Negative buffer controls were prepared concurrently to monitor for possible cross-contamination during gDNA extractions. Quantity and quality of gDNA were measured using the ND-1000 spectrophotometer (NanoDrop technologies).

*P. carinii* gDNA was detected by TD-PCR using pAZ102-E and pAZ102-H primers [30], amplifying a portion of the multicopy gene encoding the mitochondrial large-subunit ribosomal RNA (mtLSUrRNA). Nested-PCR with internal primers pAZ102-X and pAZ102-Y [31] was used when the number of *P. carinii* organisms in the samples was expected to be low. 

DNA amplification was carried out from 100 ng of a gDNA sample in a 50-µL reaction mixture containing final concentrations of 75 mM Tris-HCl (pH 8.8), 20 mM (NH_4_)_2_SO_4_, 0.01% (vol./vol.) Tween 20, 3 mM MgCl_2_, 400 µM (each) deoxynucleoside triphosphate, 1 µM (each) oligonucleotide primer (Eurogentec), and 0.02 U.µL^−1^ of Goldstar^TM^ DNA polymerase (Eurogentec). In the second round of DNA amplification, 2 µL of the first-round PCR product were used in a final volume of 50 µL; final concentrations of reagents in the PCR mixture were the same as above. DNA amplification was carried out on a PTC 200 thermocycler (MJ Research) and cycling conditions were as follows: (i) a TD-PCR [32] was performed as a first round amplification: 10 cycles comprising a denaturation step (94°C for 1 min 30 s), an annealing step (65°C to 55°C for 1 min 30 s, 1°C decrease per cycle), and an extension step (72°C for 2 min) were followed by 30 cycles comprising a denaturation step (94°C for 1 min 30 s), an annealing step (55°C for 1 min 30 s), and an extension step (72°C for 2 min), and (ii) the second round of amplification (nPCR) was performed as described by 5. Negative controls with no added gDNA as well as positive control with known *P. oryctolagi* gDNA were included in each amplification run. gDNA extraction, preparation of mix reagents, and pipeting of 1^st^-round PCR products were all performed in separate rooms to prevent possible contamination. Purified PCR products were sent for direct sequencing (GenoScreen, Lille, France). A single TD-PCR and 30 nPCR reactions per rat were performed to detect *P. carinii* gDNA in seeder and receiver rat lungs, respectively. For sentinel rats, the screening strategy was the same as for receiver rats (Figure 4). 

### Statistical analysis

*Pneumocystis* loads were expressed as the mean ± SD for each group. All comparisons were analyzed using the Mann-Whitney *U*-test. Statistical analyses were performed using the SPSS software package (version 16.0, SPSS Inc., Chicago, IL, USA). A *P*-value ≤ 0.05 was considered to be significant.
